# Non-Mono-Exponential Analysis of Diffusion-Weighted Imaging for Treatment Monitoring in Prostate Cancer Bone Metastases

**DOI:** 10.1038/s41598-017-06246-4

**Published:** 2017-07-19

**Authors:** Carolin Reischauer, René Patzwahl, Dow-Mu Koh, Johannes M. Froehlich, Andreas Gutzeit

**Affiliations:** 1 Institute of Radiology and Nuclear Medicine, Clinical Research Unit, Hirslanden Hospital St. Anna, Lucerne, Switzerland; 2 Institute for Biomedical Engineering, ETH and University of Zurich, Zurich, Switzerland; 3 Department of Radiology, Cantonal Hospital Winterthur, Winterthur, Switzerland; 4 Academic Department of Radiology, Royal Marsden NHS Foundation Trust, Sutton Surrey, UK; 5 CR-UK and EPSRC Cancer Imaging Centre, Institute of Cancer Research, Sutton Surrey, UK; 6 Department of Chemistry and Applied Biosciences, ETH Zurich, Zurich, Switzerland; 7 Department of Radiology, Paracelsus Medical University Salzburg, Salzburg, Austria

## Abstract

Diffusion-weighted imaging quantified using the mono-exponential model has shown great promise for monitoring treatment response in prostate cancer bone metastases. The aim of this prospective study is to evaluate whether non-mono-exponential diffusion models better describe the water diffusion properties and may improve treatment response assessment. Diffusion-weighted imaging data of 12 treatment-naïve patients with 34 metastases acquired before and at one, two, and three months after initiation of antiandrogen treatment are analysed using the mono-exponential, the intravoxel incoherent motion, the stretched exponential, and the statistical model. Repeatability of the fitted parameters and changes under therapy are quantified. Model preference is assessed and correlation coefficients across times are calculated to delineate the relationship between the prostate-specific antigen levels and the diffusion parameters as well as between the diffusion parameters within each model. There is a clear preference for non-mono-exponential diffusion models at all time points. Particularly the stretched exponential is favoured in approximately 60% of the lesions. Its parameters increase significantly in response to treatment and are highly repeatable. Thus, the stretched exponential may be utilized as a potential optimal model for monitoring treatment response. Compared with the mono-exponential model, it may provide complementary information on tissue properties and improve response assessment.

## Introduction

Advanced prostate cancer metastasizes to the bones with a frequency of more than 90%^1^. Up to 80% of the patients that are treated with antiandrogen therapy initially respond well with a progression-free interval of 23–37 months^2^. After this time, patients develop resistance to androgen deprivation, which inevitably leads to tumour progression^3–5^. Standard imaging techniques such as technetium-99 m bone scintigraphy and computed tomography that are used to assess the presence of bone metastases fail to accurately evaluate biological activity over time^6^. As a consequence, the response evaluation criteria of solid tumours (RECIST) consider bone metastases without associated soft-tissue masses as non-measurable^7^. For this reason, evaluation of treatment response in clinical practice primarily relies on measurements of the prostate-specific antigen (PSA) level, which has not been proven to be a surrogate biomarker for improved survival^8–10^. Thus, there is an unmet need for non-invasive biomarkers that allow assessment of treatment response in bone metastases.

Several studies have demonstrated the potential of diffusion-weighted imaging (DWI) quantified by the apparent diffusion coefficient (ADC) from the mono-exponential diffusion model for evaluating response to therapy in bone metastases from prostate cancer^11–15^. Thereby, increased values of the mean and median ADCs were observed after commencement of therapy relative to pre-treatment values. Furthermore, the results of a pilot study indicate that the evolution of ADCs over time may permit early identification of antiandrogen resistance in bone metastases^16^.

The ADC is a simple and robust quantitative biomarker but it draws an incomplete picture of the molecular motion in both healthy and pathological tissue since signal attenuation in DWI is not completely described by a mono-exponential process. For this reason, several studies have recently investigated the use of more complex non-mono-exponential diffusion models for the characterization of tumour masses^17–28^ and the evaluation of treatment response^29, 30^. The additional parameters in these models may better describe the water diffusion behaviour in bone metastases and provide complementary information on tissue properties, thus enabling more sensitive evaluation of changes in response to treatment.

Various non-mono-exponential diffusion models have been proposed to describe signal attenuation when more than two b-values are acquired. According to the intravoxel incoherent motion (IVIM) model, signal decay can be ascribed to two distinct components associated with perfusion and diffusion processes in the tissue respectively^31, 32^. Even if multiple images at lower b-values are acquired, it has been shown that relatively high signal-to-noise ratios (SNRs) are required to obtain accurate and precise estimates of the perfusion-related parameters^33, 34^. By fitting the higher b-values only, a perfusion-insensitive ADC* can be obtained since perfusion dominantly affects signal decay at lower b-values^35^. The stretched exponential model describes signal attenuation due to diffusion as a continuous distribution of sources decaying at different rates which may arise from multiple, separable pools of water molecules with different diffusion coefficients within each voxel^36^. Lower values of the stretching parameter α in this model indicate heterogeneity of the diffusion coefficients^36^. The statistical model describes the distribution of diffusion coefficients within a voxel using a truncated Gaussian distribution characterized by the position of its maximum and its distribution width. Other non-mono-exponential diffusion models include the kurtosis model^37^ and models which are based on anomalous diffusion^38^, fractal models^39^, and fractional order calculus^40^.

The aim of this exploratory study is to compare the performance of the conventional mono-exponential with various non-mono-exponential diffusion models (the IVIM, the stretched exponential, and the statistical model) for treatment monitoring in prostate cancer bone metastases. To this end, repeatability of the diffusion parameters and their changes under therapy are evaluated. Furthermore, Pearson’s correlation coefficients across times are calculated to delineate the relationship between the PSA and the fitted diffusion parameters. Beyond that, bivariate correlations between the ADC and the diffusion coefficients of each non-mono-exponential model as well as between the diffusion parameters within each model are computed to assess whether the additional parameters in the non-mono-exponential models provide complementary information on tissue characteristics that may aid treatment response assessment. Finally, model preference is investigated by evaluating the Bayesian information criterion (BIC).

## Results

### Pre-treatment values and measurement repeatability

The average pre-treatment values of the various diffusion parameters are summarized in Table 1. With regard to the mono-exponential model, the results corroborate well with literature values^11–15, 41^. Two-sided paired t-tests showed no systematic differences between the pre-treatment values (p > 0.3 for all diffusion parameters). Inter-patient variability of the parameters was below 20% for all diffusion parameters with the exception of σ* from the statistical model, and f, f·D*, and D* from the IVIM model (Table 1).

**Table 1:** Average pre-treatment values of the various diffusion parameters (all units mm^2^/s except where shown) as well as inter-patient variability, and coefficient of variation as percentage.

Model	Parameter	Average baseline value (·10^−3^ mm^2^/s)	Inter-patient variability (%)	Coefficient of variation (%)
Mono-exponential	ADC	0.76	15.1	4.4
	ADC*	0.62	14.1	5.0
IVIM	D	0.57	14.9	7.8
	D*	8.01	60.0	42.5
	f (no units)	0.14	27.5	19.8
	f·D*	1.34	51.0	20.4
Stretched exponential	DDC	0.65	18.6	5.1
	α (no units)	0.76	12.3	5.0
Statistical	D_s_*	0.97	19.9	6.4
	σ*	0.87	24.1	10.0

**Figure 1:**
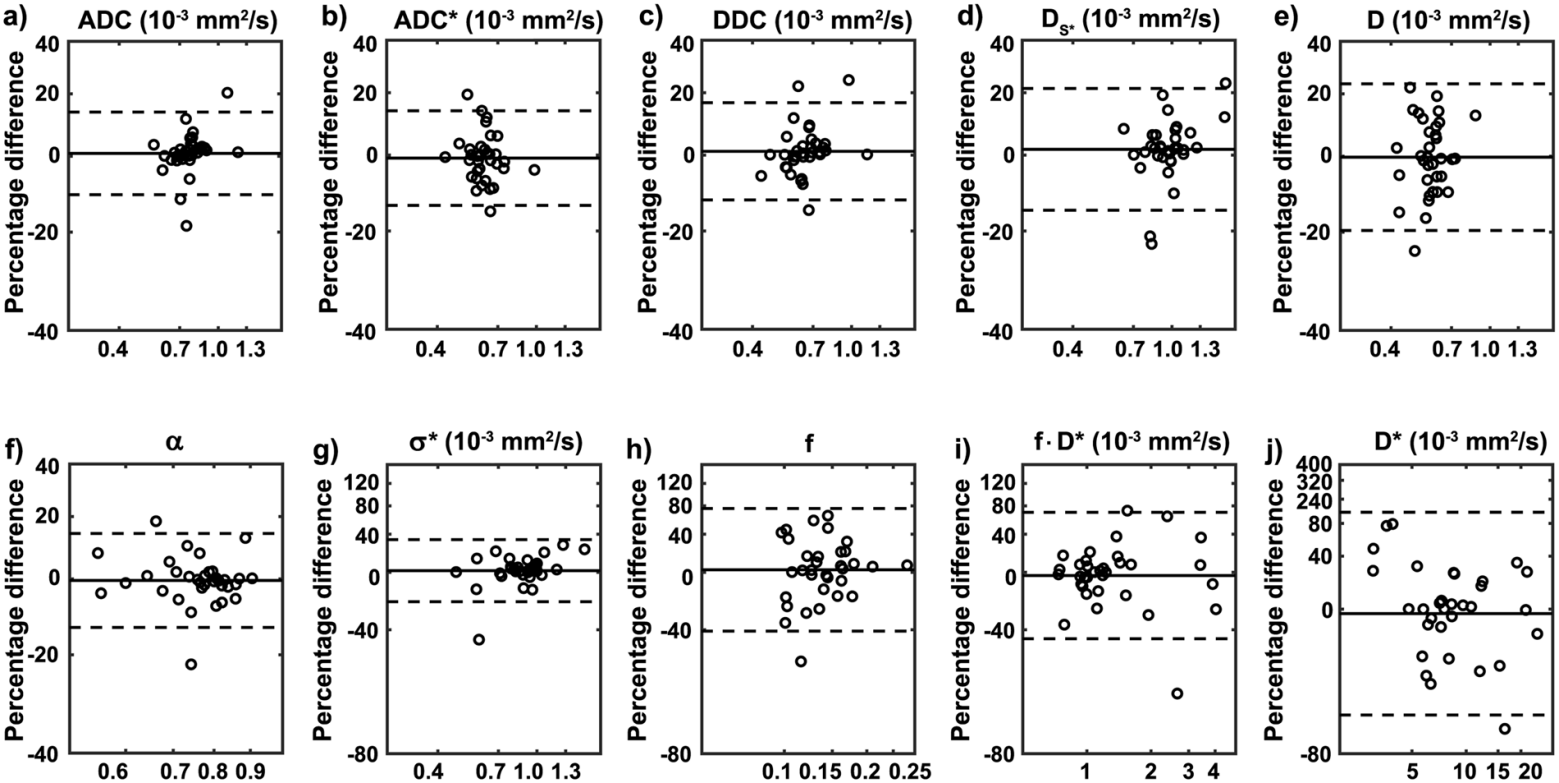
Illustration of measurement repeatability of the various diffusion parameters. Bland-Altman plots on a log-log scale of (**a**) ADC, (**b**) ADC*, (**c**) DDC, (**d**) D_s_*, (**e**) D and of the diffusion parameters (**f**) α, (**g**) σ*, (**h**) (**f**,**i**) f·D*, and (**j**) D*. The solid lines correspond to the mean differences between two estimates and the dashed lines show the 95% limits of agreement. To facilitate comparison, the same scales have been used on the y-axes of graphs (**a**–**f**) and (**g**–**i**).

Repeatability of the diffusion parameters calculated from two pre-treatment measurements indicated coefficients of variation (CVs) smaller than or equal to 10% for all parameters of the mono-exponential, the stretched exponential, and the statistical model. From the IVIM model, only D resulted in a CV below 10% with f, f·D*, and in particular D* featuring larger CVs (Table 1). These results are in agreement with previous studies in other tumours^25, 30^. Repeatability of the diffusion parameters is illustrated in Fig. 1 using Bland-Altman plots.

### Parameter changes under therapy

**Figure 2:**
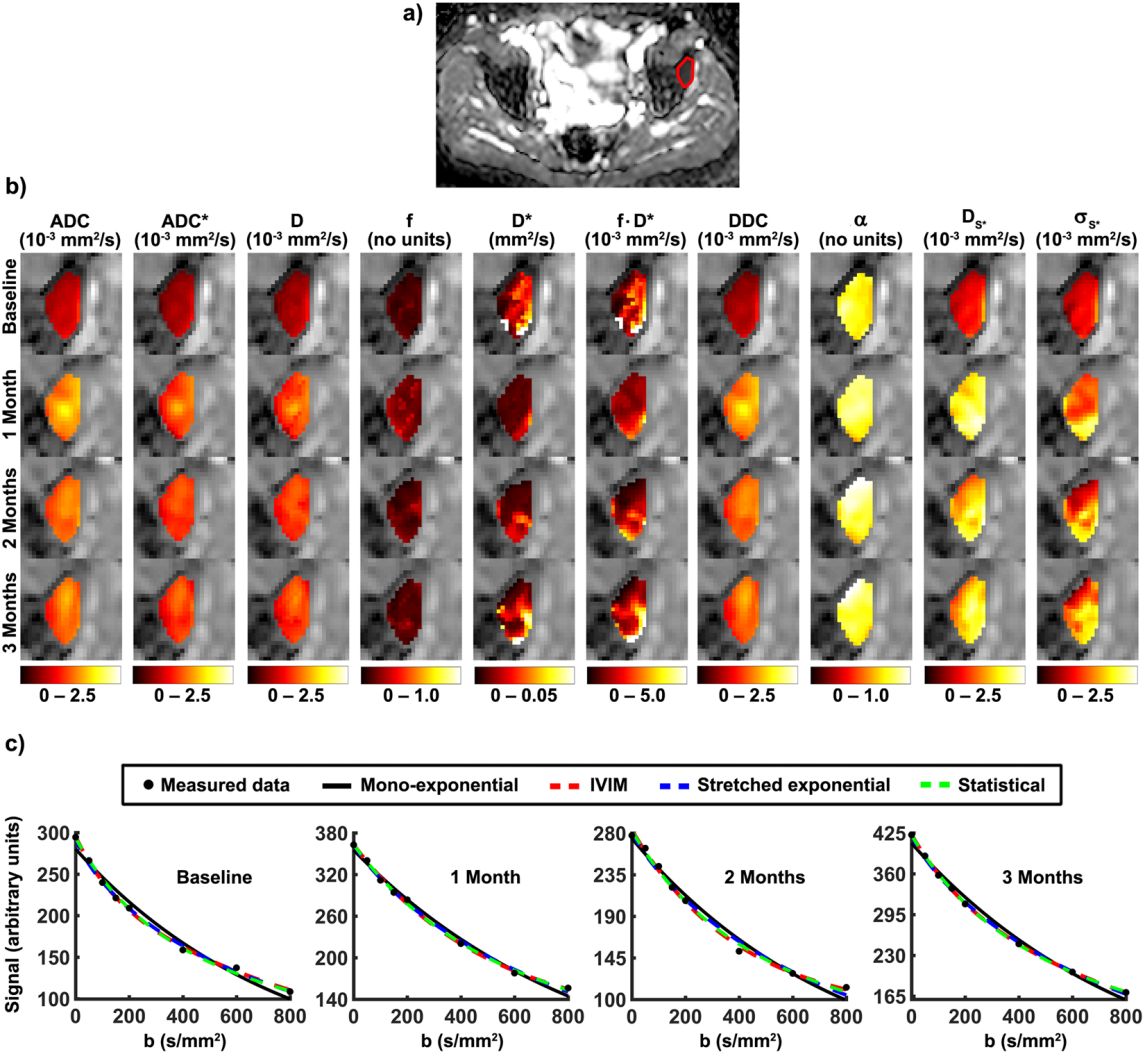
Parameter maps for an exemplary patient with a pelvic bone metastasis from prostate cancer: (**a**) ROI circumscribing the lesion superimposed onto the pre-treatment ADC map, (**b**) enlarged sections showing the parameter maps of the various diffusion parameters before therapy and one, two, and three months after treatment onset, and (**c**) measured signals and fitted curves of one voxel within the lesion at each time point.

By way of example, Fig. 2 depicts diffusion parameter maps and curve fits from one voxel within a pelvic lesion before androgen deprivation and one, two, and three months after treatment onset. The manually defined region of interest (ROI) is also depicted. There is a high similarity between the maps of the various diffusion coefficients ADC, ADC*, D, DDC, and Ds*. Visual inspection reveals an increase in the diffusion coefficients, in α from the stretched exponential, and in σ* from the statistical model under therapy. Example curve fits from one voxel within the lesion feature clear non-mono-exponential signal attenuation.

Repeated measures analysis of variance (ANOVA) revealed a significant increase in the median ADC (p < 1 · 10^−6^) and ADC* values (p < 1 · 10^−6^) under therapy. Furthermore, a significant increase in D (p < 1 · 10^−6^), f (p = 2 · 10^−6^), and f·D* (p = 2.7 · 10^−3^) from the IVIM model, in DDC (p < 1 · 10^−6^) and α (p = 0.028) from the stretched exponential, and in D_s_* (p < 1 · 10^−6^) and σ* (p < 1 · 10^−6^) from the statistical model was observed. The only parameter that showed no significant change under therapy was D* (p = 0.463) from the IVIM model. Table 2 details the average treatment changes of the diffusion parameters at each time point and the results of the pairwise comparisons of pre-treatment and post-treatment values. With regard to the mono-exponential model, the percentage changes are in agreement with literature values^12, 13, 15^.

**Table 2:** Percentage changes of the various diffusion parameters observed one, two, and three months after treatment begin. The values in parentheses are the p-values of the post-hoc comparisons of the baseline values with those one, two, and three months after anticancer treatment. Significant statistics have p < 0.017, incorporating a correction for multiple comparisons.

Model	Parameter	Mean treatment changes (%)		
		One month after treatment begin	Two months after treatment begin	Three months after treatment begin
Mono-exponential	ADC	35.2 (<1·10^−6^*)	36.3 (<1·10^−6^*)	34.6 (<1·10^−6^*)
	ADC*	35.6 (<1·10^−6^*)	35.0 (<1·10^−6^*)	28.7 (<1·10^−6^*)
IVIM	D	31.0 (<1·10^−6^*)	30.5 (<1·10^−6^*)	22.3 (8.1·10^−4^*)
	D*	12.0 (0.293)	18.2 (0.168)	14.0 (0.368)
	f	29.5 (5.2·10^−4^*)	29.5 (1.5·10^−5^*)	37.9 (4.7·10^−5^*)
	fD*	14.0 (0.073)	13.0 (0.119)	29.1 (2.6·10^−3^*)
Stretched exponential	DDC	41.5 (<1·10^−6^*)	42.9 (<1·10^−6^*)	39.6 (<1·10^−6^*)
	α	5.0 (0.005*)	4.6 (0.015*)	1.6 (0.523)
Statistical	D_s_*	38.2 (<1·10^−6^*)	39.0 (<1·10^−6^*)	37.2 (<1·10^−6^*)
	σ*	29.7 (<1·10^−6^*)	29.0 (<1·10^−6^*)	29.1 (<1·10^−6^*)

Statistically significant values are designated by an asterisk.

### Changes in PSA levels and correlations with diffusion parameters

Changes in serum PSA levels under therapy confirmed treatment response in all patients. Repeated measures ANOVA revealed a significant decrease of the PSA values under therapy (p < 0.001). Compared with pre-treatment values, the PSA decreased significantly after one (p < 0.001), two (p < 0.001), and three (p < 0.001) months. After commencement of androgen deprivation treatment, average PSA values decreased by 85.7% (range = 51.4%–98.8%) at one month, 95.2% (range = 72.4%–99.9%) at two months, and 96.6% (range = 83.5%–99.9%) at three months.

Bivariate correlation showed a moderate negative relationship between the serum PSA values and ADC, ADC* from the mono-exponential, f from the IVIM, and DDC from the stretched exponential model (Table 3). The strongest correlation thereby occurred between the PSA values and f from the IVIM model. A weak negative correlation was observed between the PSA levels and D from the IVIM and D_s_* from the statistical model (Table 3).

**Table 3:** Pearson’s correlation coefficients and corresponding p-values between the PSA and each of the various diffusion parameters.

Model	Parameters	Pearson’s correlation coefficient	p-value
Mono-exponential	ADC, PSA	−0.45	0.001*
	ADC*, PSA	−0.39	0.006*
IVIM	D, PSA	−0.29	0.044*
	D*, PSA	0.13	0.40
	f, PSA	−0.48	0.002*
	f·D*, PSA	−0.25	0.12
Stretched exponential	DDC, PSA	−0.46	0.001*
	α, PSA	−0.13	0.426
Statistical	D_s_*, PSA	−0.39	0.006*
	σ*, PSA	−0.21	0.21

Statistically significant values are designated by an asterisk.

### Parameter correlations between the various diffusion parameters

Table 4 summarizes the Pearson’s correlation coefficients and corresponding p-values between the various diffusion parameters. A very strong positive correlation was found between the ADC and the diffusion parameters from the non-mono-exponential models (Table 4) which agrees well with the similarities of the parameter maps observed in Fig. 2. Within the diffusion models a very strong positive correlation was observed between D_s_* and σ* from the statistical model. A moderate positive correlation was found between DDC and α from the stretched exponential model and a moderate positive correlation was observed between f and D* from the IVIM model. Finally, bivariate correlation showed a weak relationship between D and f·D* from the IVIM model.

**Table 4:** Pearson’s correlation coefficients and corresponding p-values between the various diffusion parameters.

Model	Parameters	Pearson’s correlation coefficient	p-value
Mono-exponential	ADC*, ADC	0.96	<1 · 10^−6^*
IVIM	D, ADC	0.86	<1 · 10^−6^*
	D, f	0.10	0.25
	D, D*	0.09	0.29
	D*, f	−0.40	3 · 10^−6^*
	D, f·D*	0.17	0.049*
Stretched exponential	DDC, ADC	0.99	<1 · 10^−6^*
	DDC, α	0.50	<1 · 10^−6^*
Statistical	D_s_*, ADC	0.98	<1 · 10^−6^*
	D_s_*, σ*	0.82	<1 · 10^−6^*

Statistically significant values are designated by an asterisk.

### Model preferences

Model preference at each time point is summarized in Table 5. Non-mono-exponential models were preferred by 85% of the tumours before treatment begin and by 97%, 97%, and 91% after commencement of antiandrogen treatment. BIC analysis revealed that the stretched exponential model was overall favoured by the majority of approximately 60% of tumours at all time points. When considering the tumours where each model was preferred, the percentage of voxels within the lesion that favoured that model ranged between 28% and 85% (Table 5). This shows that across the lesions a moderate to large percentage of voxels favoured models other than the dominant model.

**Table 5:** Number of lesions at every time point where the respective diffusion models was preferred based on the majority of voxels. The values in parentheses are the median and range percentage of voxels where the respective model was preferred, considering only lesions where the tumour model was overall favoured.

Model	Percentage of lesions where model was preferred			
	Before treatment	One month after treatment begin	Two months after treatment begin	Three months after treatment begin
Mono-exponential	5 (43; 32–50)	1 (53; 53–53)	1 (83; 83–83)	3 (44; 33–59)
IVIM	8 (43; 40–64)	5 (44; 37–79)	4 (42; 34–47)	5 (45; 33–53)
Stretched exponential	21 (42; 28–63)	21 (42; 35–68)	20 (46; 36–73)	21 (43; 29–74)
Statistical	0 (N/A)	7 (45; 40–62)	9 (36; 33–55)	5 (47; 35–85)

## Discussion

Osteoblastic bone metastases without associated soft tissue, such as those commonly encountered in prostate cancer, are classified as non-measurable according to RECIST^7^. Thus, the establishment of a non-invasive, validated response assessment is warranted since 90% of patients with advanced prostate cancer have bone metastases as the only site of disease involvement^1^. ADC quantified using the mono-exponential model has shown promise as a potential biomarker for assessing treatment response in this patient group^11–15^. However, our current study shows that there is clear statistical preference for using non-mono-exponential diffusion models compared with the mono-exponential model in bone metastases, suggesting that these models may provide better descriptions of the biological changes with treatment. The additional parameters derived using non-mono-exponential models are sensitive to treatment changes and may provide complementary information to improve the treatment response evaluation. With the exception of D* from the IVIM model, all diffusion parameters increased significantly after initiation of anti-androgen therapy compared with pre-treatment values. The fact that the stretched exponential model provided better data fitting within the majority of tumours and that the derived parameters DDC and α were highly repeatable and sensitive to treatment effects supports its wider use in this patient cohort.

Significant increases in diffusion coefficient values were accompanied by large contemporaneous decreases in the serum PSA levels, in keeping with disease response. In this regard, the highest percentage change after initiation of androgen deprivation treatment was observed for DDC from the stretched exponential model. Bivariate correlation showed a very strong positive relationship between ADC from the mono-exponential model and the diffusion coefficients from the non-mono-exponential models, indicating that they are sensitive to the same tissue characteristics and provide similar information. As pointed out in previous studies, the mono-exponential model can be considered a conditional case of other diffusion models (i.e. when α = 1 and f = 0)^28, 30^. As a result, lesions that favour the mono-exponential model are not disadvantaged by the utilization of other models but the additional parameters of these models do not add value in such tumours. Interestingly, our study also showed an increase in f values from IVIM analysis following treatment. The mechanism for an increase in f is uncertain, but may reflect vascular normalization within tumours. However, this would require further investigations.

Previous studies in other tumour types observed no significant correlation between α and DDC from the stretched exponential model^25, 28^. By contrast, a moderate positive correlation between these parameters was observed in our study. Although the biological underpinning of α is unclear, it is likely to reflect tissue heterogeneity, which may also influence the DCC measurement. Our results suggest that these parameters may contain complementary information, which could be exploited to improve treatment monitoring in prostate cancer bone metastases. Similarly, Pearson correlation showed no significant relationship between D and either f or D* from the IVIM model, indicating that these parameter report on different tissue properties. The very strong correlation of D_s_* and σ* from the statistical model, however, suggests that these parameters largely provide the same information.

Only moderate correlations between the serum PSA values and ADC, ADC* from the mono-exponential, f from the IVIM, and DDC from the stretched exponential model were observed. The known prostate cancer heterogeneity may at least partially account for the fact that only moderate relationships could be delineated. However, with respect to the ADC, the present results are in good agreement with previous work^12^.

The repeatability of the diffusion parameters is an important consideration when evaluating different diffusion models for assessing response to therapy. In agreement with previous studies in other tumours^25, 30^, our results show that the parameters from the stretched exponential model show high precision which is comparable to the repeatability of ADC and perfusion-insensitive ADC* from the mono-exponential model. High repeatability was also observed for parameters derived from the statistical model. In agreement with previous studies in other tumours^25, 30^, the precision of the perfusion-related parameters from the IVIM model was significantly worse. This may be due to the larger number of parameters that are fitted by the model, rendering it more sensitive to measurement noise. Due to time constraints in the clinical setting, only eight different b-values were acquired, which may not provide sufficient data support to reliably estimate the perfusion-sensitive components of the IVIM model. However, the generally limited measurement repeatability of the perfusion-related estimates is a well-documented drawback of IVIM modelling^33, 34^. Our study showed that the composite parameter f·D* showed a similar repeatability as f. Inversely correlated errors of f and D* as described elsewhere^30^ were not observed in our study.

The kurtosis model^37^, which has gained popularity in recent years, was not investigated in our current work since the non-Gaussian diffusion behaviour that underlies the kurtosis model has negligible effect in our acquired b-value range^37, 42^. Kurtosis imaging relies on the acquisition of a maximum b-value of at least 1500 s/mm^2^, which is usually not incorporated in clinical body imaging^42^. The inclusion of higher maximum b-values would prolong the minimum achievable echo time, thus lowering the SNR and in turn decreasing accuracy and precision of the parameter estimates. Beyond that, higher maximum b-values increase eddy current-induced image distortions in the data.

There are limitations to our study. First, we included a relatively small patient cohort and larger patient studies would be useful to validate our findings. However, as most patients in this disease setting would have received antiandrogen, chemotherapy or radiation therapy prior to any imaging, the relatively low patient number from a single institution is not unexpected. Second, while our study findings are in agreement with other studies evaluating non-mono-exponential diffusion models in cancer^18, 21, 25, 30^, more b-values in particular at the lower end of the range may be needed to realize the full potential of IVIM modelling for treatment monitoring of bone metastases.

In conclusion, our study shows that there is a statistical model fit preference for using non-mono-exponential diffusion models in bone metastases from prostate cancer before and after initiation of antiandrogen treatment. The additional parameters derived using these models may provide additional information, which may improve the evaluation of treatment response and may permit detecting the onset of antiandrogen resistance at an earlier time point. Our results show that the stretched exponential model was favoured within the majority of tumours. Its derived parameters DDC and α demonstrated very good measurement repeatability and were also sensitive to treatment effects. We observed a moderate correlation of the two parameters, which indicates that they contain a certain amount of complementary information, which may be exploited to improve treatment response assessment. Thus, the stretched exponential may be utilized as a potential optimal model for monitoring treatment response in prostate cancer bone metastases.

## Methods

### Patient population

This prospective, single-centre clinical study was approved by the Cantonal Research Ethics Committee (Zurich, Switzerland) and written informed consent was obtained from all patients. This study was conducted in accordance with the Declaration of Helsinki. Seventeen treatment-naïve patients with prostate cancer bone metastases who fulfilled all inclusion and exclusion criteria were included. The inclusion criteria were as follows: adults with histologically proven prostate cancer, evidence of bone metastases in the pelvis confirmed on skeletal scintigrams and no prior history of antiandrogen treatment, chemotherapy or radiation therapy. The exclusion criteria were as follows: history of another malignancy, contraindication to MRI or unwillingness to participate in the clinical study. Five patients had to be secondarily excluded: three patients deceased prior to termination of the study and two patients withdrew willingness to participate in the study after an acute deterioration in their health condition. Thus, twelve men (mean age = 76, range = 67–85) with a total of 34 pelvic bone metastases from prostate cancer (mean size = 7.64 cm^3^, range = 2.64–39.65 cm^3^) were included in the analysis.

### Diagnosis of bone metastases at skeletal scintigraphy

Diagnosis of metastatic disease in the pelvis was made within seven days of the baseline MRI examination using whole-body skeletal radionuclide scintigraphy, which was performed three hours after injection of 550 Mbq ^99m^Tc-labeled dicarboxypropane diphosphonate (Tecleos, Cisbio, Gif-sur-Yvette, France). Images were acquired using a dual-head whole-body scanner with a low energy high-spatial-resolution collimator (E-CAM, Siemens, Munich, Germany).

### Androgen deprivation or androgen ablation treatment

All patients underwent androgen deprivation by means of either medical treatment (three patients received goselerin (Zoladex^®^), two patients leuprorelin acetate (Lucrin^®^), two patients goselerin (Zoladex^®^) + denosumab (Xgeva^®^), two patients leuprorelin acetate (Lucrin^®^) + denosumab (Xgeva^®^), and one patient goselerin (Zoladex^®^) + bicalutamide (Casodex^®^) + denosumab (Xgeva^®^)) or surgical orchiectomy (two patients). Antiandrogen treatment was commenced within two days of the baseline MRI examination.

### MRI acquisition

Image acquisition was performed on a 1.5 T whole-body scanner (Achieva, release 3.2.3.4, Philips Healthcare, Best, the Netherlands) within two days before and repeated one, two, and three months after the commencement of antiandrogen treatment. Pelvic images were acquired with the patient in the supine position by using a four-element receive-only body coil array (Philips, Healthcare, Best, the Netherlands). The imaging protocol consisted of transversal T_1_-weighted, T_2_-weighted, and proton density-weighted sequences followed by axial DWI including eight b-factors (b = 0, 50, 100, 150, 200, 400, 600, 800 s/mm^2^). Details of the MRI sequences are summarized in Table 6. The total measurement time of the protocol amounted to approximately 20 minutes. At baseline, the DWI scan was acquired twice to permit assessment of measurement repeatability.

**Table 6:** Magnetic resonance imaging sequence parameters.

Sequence	Repetition Time (ms)	Echo Time (ms)	No. of Signal Averages	Acquisition time (s)
SPIR T1-weighted fast SE	1000	3.7	4	208
T2-weighted fast SE	2850	80	4	262
Proton density–weighted fast SE	2850	4.7	4	262
DWI with free-breathing SE echo-planar imaging and SPIR*	4506	63	5	640

SE = spin echo, SPIR = spectral presaturation with inversion recovery.

All sequences were axial and two-dimensional (number of slices = 32, slice thickness = 6 mm, field-of-view = 400 × 256 mm^2^, voxel size = 2 × 2 mm^2^).

*This sequence was performed with b values of 0, 50, 100, 150, 200, 400, 600, and 800 s/mm^2^ and a parallel imaging reduction factor of 1.6.

### Serum PSA measurements for treatment response assessment

To assess treatment response, the serum PSA levels were determined in tandem with each MRI examination. Venous blood was therefore drawn from all patients via the right or left cubital vein before and at one, two, and three months after starting androgen withdrawal treatment. A decline in PSA levels by more than 50% confirmed by a second measurement four weeks later was accepted as a response to treatment^43^.

### Diffusion data analysis

Data analysis was performed using in-house software written in MATLAB (The MathWorks, release 2016a, Natick, MA, USA). Prior to image analysis, eddy-induced image warping and motion were corrected in the *in-vivo* data sets using a correlation-based affine registration algorithm^44^. Parameter maps were calculated by least-squares fitting using the mono-exponential and three non-mono-exponential diffusion models.

Estimates of the ADC were computed by mono-exponential fitting of the DWI data using the following equation:

1 $$S(b)={S}_{0}\,{\exp }(-bADC),$$

where *S*(*b*) corresponds to the signal with and *S*_*0*_ without diffusion weighting and *b* denotes the b-value. To diminish the influence of perfusion effects, a mono-exponential fit of all DWI data with b > 200 s/mm^2^ was also performed (ADC*).

Estimates of D, D*, and f were obtained by fitting the IVIM model to the DWI data^31, 32^:

2 $${S}({b})={S}_{0}({fexp}(-b{D}^{\ast })+(1-f)\exp (-bD)),$$

with 0 ≤ f ≤ 1. In addition, the composite parameter f·D* was also computed which was shown to be linked to the relative perfusion or blood flow^45^. Starting values of D, D*, and f were computed by a least-squares fit of a mono-exponential curve to the DWI data with b > 200 s/mm^2^ followed by another mono-exponential curve fitted to the remaining signal at the lower b-values^28^.

Estimates of DDC and α were determined by fitting the stretched exponential model to the DWI data^36^:

3 $${\rm{S}}({\rm{b}})={{\rm{S}}}_{0}\exp (-{({\rm{bDDC}})}^{{\rm{\alpha }}})$$

with 0 ≤ α ≤ 1.

Estimates of D_s_ and σ were computed by fitting the statistical model to the DWI data^46^:

4 $$S(b)={S}_{0}(\frac{1+\varphi (\frac{{D}_{S}}{\sigma \sqrt{2}}+\frac{b\sigma }{\sqrt{2}})}{1+\varphi (\frac{{D}_{S}}{\sigma \sqrt{2}})})\exp (-b{D}_{S}+\frac{1}{2}{b}^{2}{\sigma }^{2}).$$

Based on previously published results^47^, the parameters were reformulated for statistical analysis to obtain the mean (D_s_*) and the standard deviation (σ*) of the distribution of diffusion coefficients within a voxel^28^.

The parameter maps acquired one, two, and three months after the commencement of androgen withdrawal therapy were co-registered to the corresponding pre-treatment parameter map for each patient. For this purpose, each post-treatment ADC map was co-registered to the corresponding pre-treatment ADC map using a robust multiresolution alignment algorithm^48^ that was previously extended to allow for affine transformations^12^. The resulting transformation matrix was subsequently used to co-register the corresponding post- and pre-treatment parameter maps of the other diffusion parameters.

In line with previous work^12, 16^, ROIs were manually defined on the baseline ADC maps, taking into account the diagnostic information of the skeletal scintigrams and the corresponding conventional anatomical images to simplify lesion localization. For each metastasis, ROIs were circumscribed on the transversal plane of each tumour-bearing slice and subsequently summed up, resulting in a three-dimensional ROI enclosing the entire lesion. Thereafter, the ROIs were applied to subsequent scans and to the corresponding parameter maps obtained using the non-mono-exponential diffusion models. Median values of each diffusion parameter were calculated in order to reduce sensitivity to outlier values and since the parameters were not normally distributed, resulting in ten measures at each time point. In patients with multiple lesions, the weighted median value of each diffusion parameter was also calculated on a per-patient basis in order to permit assessment of correlations with the PSA values.

### Statistical analysis

Statistical analysis of the data was performed with SPSS (Statistics for Windows, version 23.0, IBM, Armonk, NY, USA). All statistics was calculated on the log-transformed data, back transformations were performed where appropriate^49^.

Using the two pre-treatment measurements of each diffusion parameter, measurement repeatability was assessed with the method of Bland and Altman^50^. Repeatability of the parameters was quantified using the CV defined as $CV=100 \% \cdot \sqrt{\exp ({\sigma }^{2}/2)-1}$, where *σ* ^2^ corresponds to the variance of the difference between the baseline parameters. Differences in the pre-treatment values of the parameters were assessed using two-sided paired t-tests.

Cohort baseline values were computed as the mean of the logarithm of the two baseline measurements. Inter-patient variability was assessed by computing the variance of the mean of the logarithm of the two baseline values across all patients. The data were back transformed to percentages using $100 \% \cdot \sqrt{\exp ({\sigma }^{2})-1}$. Changes in the PSA values and the diffusion parameters of the four diffusion models under therapy were analysed using repeated measures ANOVA followed by pairwise comparisons of the parameter values before and after beginning of treatment. Using the Bonferroni correction, the significance level was set at p < 0.017 to adjust for multiple comparisons. In all other statistical analyses, a p-value of p < 0.05 was considered statistically significant. In these cases, no corrections for multiple comparisons were performed and raw p-values are reported since this study is primarily descriptive and many of the parameters are expected to exhibit a high degree of correlation.

Pearson’s correlation coefficient across times was used to assess correlations between the PSA and the parameters of each diffusion model. Moreover, correlations between the ADC and the diffusion coefficients of the non-mono-exponential models and between the parameters within each non-mono-exponential model were evaluated.

Statistical preference for the four models in the lesions was quantified at each time point using the BIC which penalizes for model complexity^51^:

5 $$BIC=-2logL(\theta )+klog(n),$$

where *L(θ)* denotes the value of the maximized likelihood objective function for a model with *k* parameters and *n* corresponds to the number of data points. In each voxel, the preferred model was given by the model with the lowest value of the BIC. The majority vote across all voxels of each lesion was used as preferred model within each lesion.
